# Anomaly in the Chain Length Dependence of *n*-Alkane Diffusion in ZIF-4 Metal-Organic Frameworks

**DOI:** 10.3390/molecules23030668

**Published:** 2018-03-15

**Authors:** Seungtaik Hwang, Arun Gopalan, Maximilian Hovestadt, Frank Piepenbreier, Christian Chmelik, Martin Hartmann, Randall Q. Snurr, Jörg Kärger

**Affiliations:** 1 Faculty of Physics and Earth Sciences, Universität Leipzig, Linnéstraße 5, 04103 Leipzig, Germany; seungtaik.hwang@physik.uni-leipzig.de (S.H.); chmelik@physik.uni-leipzig.de (C.C.); 2 Department of Chemical and Biological Engineering, Northwestern University, 2145 Sheridan Road, Evanston, IL 60208-3109, USA; arungopalan2020@u.northwestern.edu (A.G.); snurr@northwestern.edu (R.Q.S.); 3 Erlangen Catalysis Resource Center, Friedrich-Alexander-Universität Erlangen-Nürnberg, Egerlandstraße 3, 91058 Erlangen, Germany; maximilian.hovestadt@fau.de (M.H.); frank.piepenbreier@fau.de (F.P.); martin.hartmann@ecrc.uni-erlangen.de (M.H.)

**Keywords:** ZIF-4, *n-*alkanes, transport diffusivity, commensurate/incommensurate adsorption, GCMC simulation

## Abstract

Molecular diffusion is commonly found to slow down with increasing molecular size. Deviations from this pattern occur in some host materials with pore sizes approaching the diameters of the guest molecules. A variety of theoretical models have been suggested to explain deviations from this pattern, but robust experimental data are scarcely available. Here, we present such data, obtained by monitoring the chain length dependence of the uptake of *n-*alkanes in the zeolitic imidazolate framework ZIF-4. A monotonic decrease in diffusivity from ethane to *n-*butane was observed, followed by an increase for *n-*pentane, and another decrease for *n-*hexane. This observation was confirmed by uptake measurements with *n-*butane/*n*-pentane mixtures, which yield faster uptake of *n-*pentane. Further evidence is provided by the observation of overshooting effects, i.e., by transient *n-*pentane concentrations exceeding the (eventually attained) equilibrium value. Accompanying grand canonical Monte Carlo simulations reveal, for the larger *n-*alkanes, significant differences between the adsorbed and gas phase molecular configurations, indicating strong confinement effects within ZIF-4, which, with increasing chain length, may be expected to give rise to configurational shifts facilitating molecular propagation at particular chain lengths.

## 1. Introduction

Among numerous recent studies of metal-organic frameworks (MOFs), a large portion of them have been focused on zeolitic imidazolate frameworks (ZIFs) due to their structural flexibility and high thermal and chemical stability, which are important for applications in gas separation and storage [1,2,3,4]. In particular, ZIF-4, which exhibits remarkable structural flexibility and undergoes a phase transition with temperature variations, has attracted great attention [5,6]. The significant potential of ZIF-4 for adsorptive separation of olefins and paraffins was already studied [4], and its new scale-up synthesis route to become a commercial adsorbent for gas separation processes was recently reported in literature [7]. 

The present study reports, with infrared microscopy (IRM), a non-monotonic chain length dependence of the transport diffusivities of *n-*alkanes in ZIF-4. First claims of having observed anomalies of this type with *n-*alkanes in microporous materials date back to 1973 when R.L. Gorring recorded molecular uptake rates of *n*-alkanes from ethane to tetradecane in beds of crystallites of zeolite T [8]. The diffusivities resulting from his analysis were found to monotonically decrease from ethane to octane over more than two orders of magnitude, followed by an increase up to dodecane over two orders before, with further increasing chain length, the diffusivities once again continued to decrease. With reference to the “window” in the carbon numbers, over which the normally expected behavior appeared to be interrupted, the (seeming) anomaly has become known under the term “window effect”. This term is still in use, even if the postulated effect is today known to be an artifact, which has been caused by a number of unjustified simplifications in data analysis [9]. Subsequent, more stringent measurements did not reveal any indication of deviations from a monotonic decay of the diffusivities of *n*-alkanes in zeolite T [10,11].

Gorring’s postulation, though incorrect, triggered a most productive search for the conditions under which guest molecules give rise to diffusivities rising rather than falling with increasing chain length in microporous host systems. For the thus developed models different terms came into common use, including “resonant diffusion” [12,13], “levitation effect” [14,15], “commensurate adsorption” [16,17,18,19] and “incommensurate diffusion” [19,20,21]. The scenario of the commensurate/incommensurate adsorption and diffusion was, in particular, evidenced by configurational-bias Monte Carlo (CBMC) and grand canonical Monte Carlo (GCMC) simulations revealing the configurations of guest molecules under confinement by the host material [16,22,23,24].

All these simulations left no doubt about the possible existence of anomalies in the chain length dependence of guest diffusion in microporous host materials and that, given the appropriate system, these anomalies should be experimentally accessible. Attaining such experimental evidence, however, is complicated by the fact that diffusion measurement with microporous materials is, as a rule, subject also to influences different from diffusion [25,26] impeding the measurement accuracy. Thus, the—most likely rather modest—effects of chain length anomalies might be within the limits of measurement accuracy. This has been the situation with diffusion measurements of *n*-alkanes in zeolite NaCaA [27] where, in pulsed-field gradient nuclear magnetic resonance (PFG NMR) measurements, the chain length dependence of the diffusivities is found to exhibit but a weak maximum around *n*-decane, with a slightly more pronounced effect observed with the diffusivities determined by quasi-elastic neutron scattering (QENS) around *n*-dodecane [28]. A related situation was met in the search for experimental evidence of the levitation effect where, in QENS diffusion studies with pentane isomers in FAU type zeolites, diffusivities could in fact be seen to increase with increasing molecular critical diameters [29] while the PFG NMR diffusivities showed the usual trend of decreasing with increasing molecular diameters [30]. Such differences in the message of QENS and PFG NMR, however, may be referred to the existence of internal transport resistances [31] by, e.g., stacking faults [32] if their mutual distances (“spacings”) are in between the diffusion path lengths recorded by QENS (nanometers) and PFG NMR (micrometers). 

With the advent of the techniques of microimaging by IR and interference [33,34] it has become possible to perform uptake experiments with the individual crystallites/particles of the material under study. In this way, microscopic diffusion measurements could be performed, for the very first time, under also non-equilibrium conditions with, essentially, no restriction in the upper limit of the observation time. In this way, deviating from PFG NMR and QENS, the range of diffusivities accessible to direct measurement can be extended to arbitrarily small values. Differing from conventional (i.e., macroscopic) uptake measurements, microimaging may be implied to remain essentially unaffected by thermal effects [35] and by any external transport resistance due to diffusion through a bed of crystals (which, in [9], have been identified as two major mechanisms affecting the experimental data in Gorring’s classic paper [8]).

We have exploited these novel options of diffusion measurements to investigate the chain length dependence of the diffusivities of C2 to C6 *n*-alkanes in MOFs of type ZIF-4. We were motivated by the prospect that the variation in compatibility between pore and guest sizes with increasing chain length might appear in variations from a monotonic decay in the diffusivities. Correspondingly, after presenting the experimental data of these measurements in Section 2, Section 3 shall report first results of molecular simulations elucidating the constraints the *n*-alkanes are subject to within MOF ZIF-4, as a function of their chain length. A detailed description of the materials and the performed experiments is provided in Section 4.

## 2. Diffusion Measurements

### 2.1. Single-Component Uptake Experiments

Uptake experiments were performed by recording the increase in loading, *m*(*t*), as resulting from the intensity of a characteristic absorption band in the IR spectrum [36] initiated by a pressure increase in the surrounding atmosphere. Analysis of the thus determined uptake curves *m*(*t*)/*m*(∞) was based on the relation [37]

(1) $$\frac{m\left(t\right)}{m\left(\infty \right)}=1-\frac{6}{\pi^{2}}\overset{\infty }{\underset{n=1}{\sum }}\frac{1}{n^{2}}\exp\left(-\frac{n^{2}\pi^{2}D_{T}t}{R^{2}}\right)$$

for diffusion-limited uptake by a sphere-like adsorbent particle of radius *R*, with $D_{T}$ denoting the diffusivity or, more specifically, the transport diffusivity being defined, via Fick’s first law

(2) $$j=-D_{T}\mathrm{grad}c$$

as the factor of proportionality between the concentration gradient of the guest molecules and the thus emerging molecular flux. Equation (1) holds under the simplifying assumption that the variation in the magnitude of the diffusivity over the concentration range covered during the uptake process is negligibly small in comparison with the diffusivity. In our experiments, such a situation was indeed cared for by choosing sufficiently small pressure steps.

**Figure 1:**
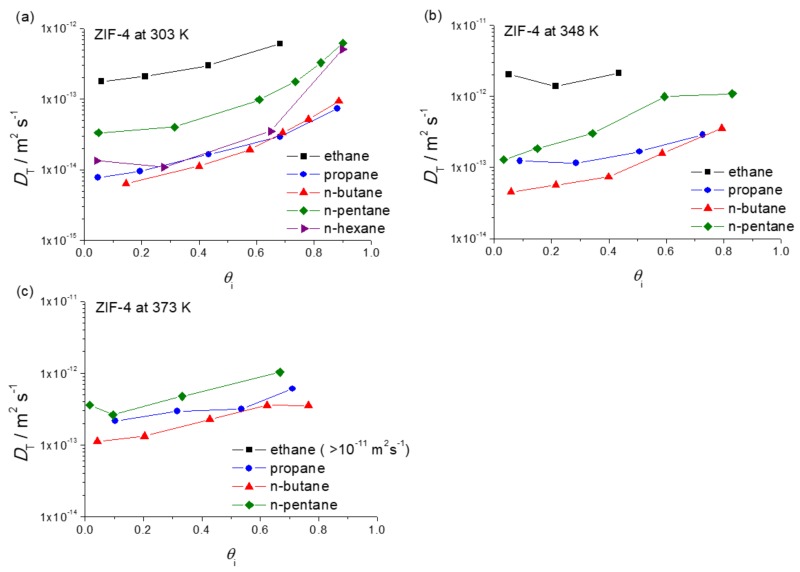
Transport diffusivities of *n*-alkanes in ZIF-4 as a function of fractional pore occupancy at (**a**) 303 K; (**b**) 348 K and (**c**) 373 K. Note that *n*-hexane was measured only at 303 K.

Figure 1 provides a survey of the thus determined diffusivities. They are plotted as a function of the fractional pore occupancy, $\theta_{i}$, for each guest molecule, which can be defined as follows [38,39,40]:

(3) $$\theta_{i}=\frac{q_{i}}{q_{i,sat}}$$

where $q_{i}$ is the molar loading of species i and $q_{i,sat}$ is the molar loading of species i at saturation.

One easily recognizes the increase in the diffusivities with increasing pore occupancy which is known to occur as a quite common pattern for diffusion within cage-type adsorbents with narrow windows [41]. The fractional pore occupancy in Figure 1 was determined based on the IR signals measured in arbitrary units. The validity of this calculation was cross-checked by comparing the adsorption isotherms (in relative units) obtained via IR microscopy (IRM) with the adsorption isotherms measured gravimetrically and volumetrically (see in Section 4.4).

Transport diffusivities may, equally obviously, be expected to increase as well with increasing temperature. This does indeed become evident on comparing the diffusivity data in Figure 1 obtained for the different temperatures considered in our study. Such a conclusion is, as a matter of course, totally correct on comparing the diffusivities at identical pore occupancies. It might be worthwhile mentioning, however, that for measurements where the external pressure (rather than the intrinsic loading) is kept constant, diffusivities may be found to even decrease with increasing temperature [42]. Such a—on first glance possibly rather strange—behavior may, however, be rationalized by the fact that, at a fixed external pressure, temperature increase leads to a dramatic decrease in pore occupancy which—as just discussed—reduces the diffusivities.

**Figure 2:**
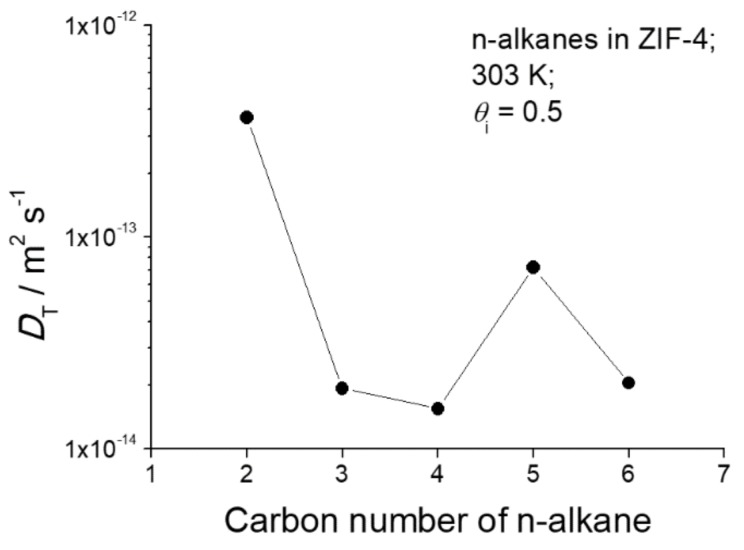
Transport diffusivities of ethane, propane, *n*-butane, *n*-pentane, and *n*-hexane at a fractional pore occupancy of 0.5 in ZIF-4 at 303 K.

As the most important message of Figure 1, the diffusivities of *n*-pentane are seen to oppose the general trend of the diffusivities of the other *n*-alkanes. Compared with propane and butane, *n*-pentane diffusion is seen to be enhanced rather than reduced, followed by a decrease in the diffusivity of *n*-hexane. A survey of this pattern at 303 K is shown in Figure 2, for diffusion measurements at loadings at about half pore occupancy. In this non-monotonic pattern or periodic rise and fall of the diffusivities with *n*-alkane chain length, we recognize the celebrated “window effect” mentioned in the introduction. It is noticeable that the diffusivity of *n*-hexane at a high pore occupancy is close to that of *n*-pentane (Figure 1a). Further investigations are required to understand how the configuration of *n*-hexane within ZIF-4 changes with increasing pore occupancy and to rationalize the enhancement of *n*-hexane diffusivity at the high pore occupancy.

### 2.2. Two-Component Uptake Experiments

**Figure 3:**
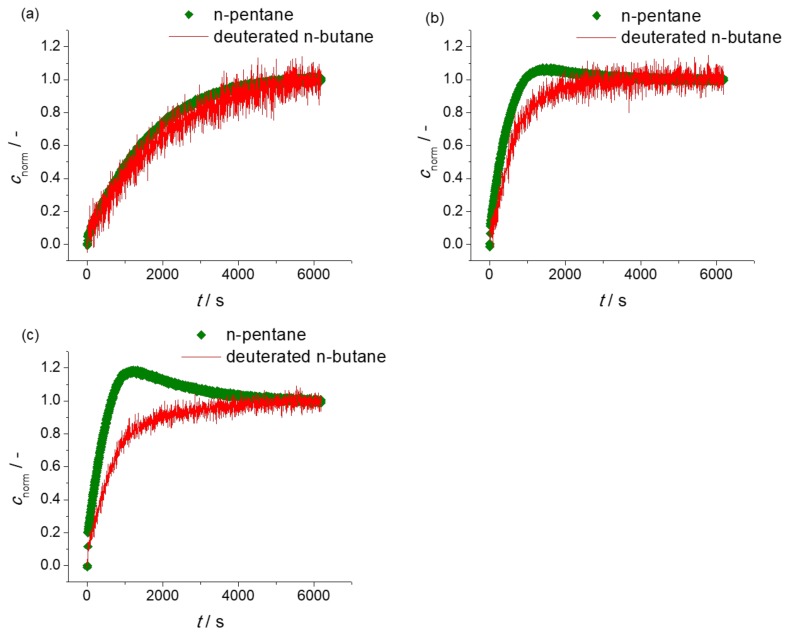
Uptake curves for binary mixture of *n*-pentane and deuterated *n*-butane at (**a**) low; (**b**) medium; (**c**) high overall pore occupancies.

In addition to “simple” uptake measurements with the individual components, IRM also provides the possibility of measuring, simultaneously, the uptake of various components in their mixture [43,44]. By this type of measurement, the uptake rates of two components may be directly compared with each other. In uptake measurements with mixtures of *n*-butane and *n*-pentane on ZIF-4, correspondingly, the rate of *n*-pentane uptake is expected to exceed that of *n*-butane. Such patterns may indeed be seen in Figure 3a showing the two-component molecular uptake with *n*-butane and *n*-pentane mixtures by ZIF-4 with relatively low partial pressures in the surrounding atmospheres and, correspondingly, also relatively small molecular concentrations. At these low loadings, molecular uptake of *n*-butane and *n*-pentane may be expected to occur essentially independent from each other.

The evidence provided by the two-component measurement may be further enhanced on considering concentration ranges high enough so that the mutual interaction between the two types of guest molecules becomes relevant. This is the case with the situations considered in Figure 3b and, to an even larger extent, in Figure 3c. Now, because of the higher partial pressure in the surrounding atmosphere and owing to its higher diffusivity, *n*-pentane is seen to attain loadings which, obviously, exceed the equilibrium value corresponding to the given partial pressures in the surrounding atmosphere.

We are now in a situation which is not correctly described anymore by simple Fick’s 1st law, Equation (2). One has rather to go back to the more general perspective of irreversible thermodynamics in which the gradient of the chemical potential of a given component (rather than the gradient of its concentration) is the driving force of diffusive fluxes. By doing so, the diffusive flux of *n*-pentane into the ZIF-4 particle is immediately seen to be driven by the concentration gradients of both *n*-pentane and *n*-butane. Thus, for sufficiently large gradients in the concentration of *n*-butane, the flux of *n*-pentane may, eventually, be seen to be directed “uphill”, that is, into the direction of increasing *n*-pentane concentrations. The thus initiated increase in pentane concentration towards the interior of the ZIF-4 particle is easily recognized as the origin of the observed “overshooting”, i.e., of the attainment of *n*-pentane concentrations above the equilibrium values. As a consequence of the continued uptake of the (slower) *n*-butane molecules, their concentration gradient towards the interior is gradually decreasing so that, in turn, more and more of the pentane molecules which have entered the ZIF-4 particles in excess are now diffusing out of the particle, until the establishment of the final, overall equilibrium. The phenomenon of overshooting is intrinsically tied to the larger diffusivity of the overshooting component and serves, therefore, as a second, independent confirmation of the observation of the window effect with *n*-alkanes in ZIF-4.

## 3. Molecular Configurations

The molecular configurations of C_2_–C_5_ *n*-alkanes adsorbed in ZIF-4 were calculated using grand canonical Monte Carlo (GCMC) simulations. See Section 4.2 for details. Although ZIF-4 is known to be quite flexible, we started with simulations in a rigid framework. Table 1 shows the predicted enthalpies of adsorption at 2 bar and 303 K. For most systems, the enthalpy of adsorption of *n*-alkanes increases in magnitude monotonically as the chain length increases. Table 1, however, shows that the binding of *n*-pentane is weaker than the binding of *n*-butane or propane, providing a first indication that *n*-pentane may not have room to fit comfortably inside the ZIF-4 cages. This could be an indirect indicator of the “window effect”.

**Figure 4:**
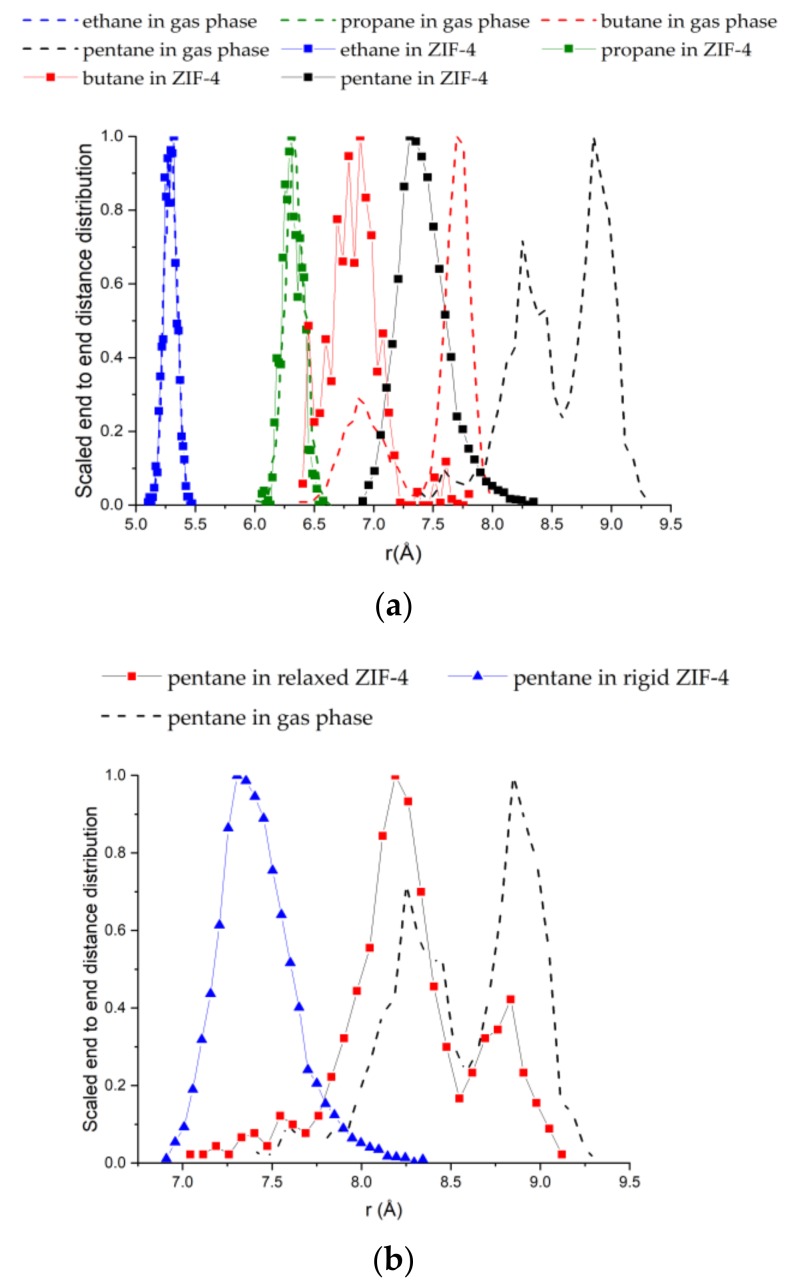
(**a**) Distributions of the end-to-end chain lengths of C2–C5 alkanes adsorbed in ZIF-4 compared with those in the gas phase. *n*-Pentane and *n*-butane molecules coil up significantly to fit inside the cages of ZIF-4; (**b**) Distributions of the end-to-end chain lengths of *n*-C5 in the gas phase, in rigid ZIF-4, and in the relaxed ZIF-4 structure from Figure 5b.

Figure 4a compares the end-to-end chain length distributions of the alkane molecules adsorbed in ZIF-4 with those in the gas phase. For reference, the fully stretched chain lengths are given in Table 2. For ethane and propane, there is no difference between the gas phase distribution and the distribution under confinement. However, there is a significant reduction in the end-to-end lengths for *n*-butane and *n*-pentane upon adsorption in ZIF-4, indicating that they must adopt coiled configurations inside the ZIF-4 cages—keeping in mind the assumption of a rigid framework.

**Figure 5:**
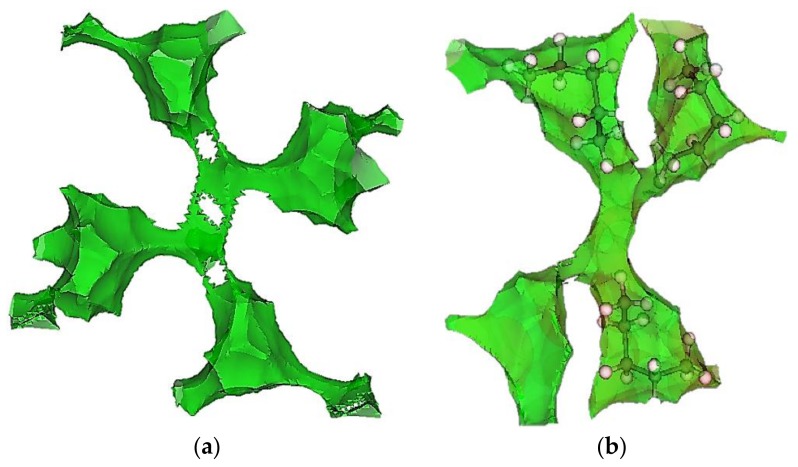
The 1 Å distance isosurface (green) for a typical pore junction in ZIF-4. (**a**) Rigid guest-free structure and (**b**) relaxed structure where three out of the four cages connected to the junction have *n*-pentane in them.

Given the tight fit of *n*-pentane in ZIF-4, it becomes important to consider the framework flexibility. In particular, we hypothesized that in a flexible lattice, *n*-pentane might stretch into the window regions of ZIF-4, and that this stretching might be responsible for the “window effect” observed in the experiments. To test this idea, we relaxed the structure of ZIF-4 at a high loading of pentane (2 bar, 303 K, ~7 molecules per unit cell). Figure 5a shows the surface of the pores (at a distance of 1 Å from the framework atoms) in the rigid structure, and Figure 5b shows the pore surface after relaxation in the presence of the *n*-pentane molecules [45]. Shown is a typical junction in ZIF-4 where three out of the four cages connected to the junction have *n*-pentane in them. It is clearly seen from Figure 5b that the windows of the cages with pentane molecules in them (three out of four) are opened while the fourth window appears to be closed, suggesting that adsorption of *n*-pentane can open the windows of ZIF-4. The distribution of end-to-end distances from GCMC in the relaxed structure (Figure 4b) looks more like the gas phase distribution and clearly indicates stretching of the alkane chains in the open structure (compared to the rigid framework), supporting our hypothesis. In future work, we will consider framework flexibility directly in the GCMC simulations. It was also found that the cages of the rigid ZIF-4 were too small to accommodate *n*-hexane, thereby predicting almost a zero loading of *n*-hexane on the current GCMC simulations. Hence, adsorption of *n*-hexane will be another subject of our future work, along with the inclusion of the framework flexibility of ZIF-4.

## 4. Materials and Methods

### 4.1. Structure of ZIF-4

ZIF-4 is made of tetrahedral Zn^2+^ nodes connected by imidazolate linkers in a CAG topology, with the nitrogen atom on the imidazolate being the site of coordination. CAG is known to have two kinds of pore regions, the “cages” (the larger cavities) and the “junctions,” which connect four different cages. Each unit cell has eight cages, each of which opens into two junctions through separate cage windows. These windows are each lined with four freely rotating imidazolate linkers, which enable the “swinging-opening” motion of the cage windows [5]. The cages in ZIF-4 have a maximum diameter of 5.14 Å, and the pore-limiting diameter at the cage windows is 2.45 Å for the guest-free framework. Some of this structural information (calculated using Zeo++ using a 2.4 Å He probe) is summarized in Table 3. Figure 5 provides a visual representation of the pore network of ZIF-4. The distance isosurfaces in Figure 5 were calculated using Zeo++ [46] before and after the adsorption of *n*-pentane (with structure relaxation in Figure 5b). The framework atoms were assigned pre-defined radii and all points which are at the same set distance to the surface of the framework were then connected to form the corresponding distance isosurface.

**Figure 6:**
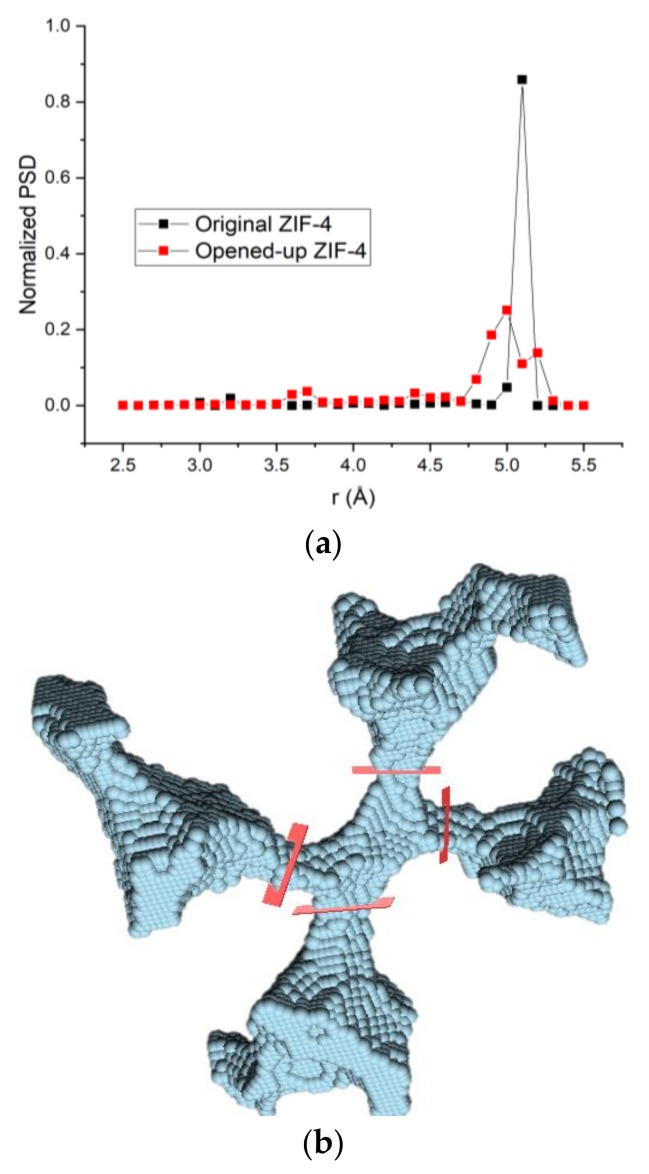
(**a**) Pore size distributions of original ZIF-4 and the “opened-up” ZIF-4 calculated using Zeo ++; (**b**) A typical pore junction (center) in the “opened-up” ZIF-4 visualized using Poreblazer (using a probe of 2 Å diameter on a 0.2 Å grid). Windows are marked for clarity in red. The “opened-up” ZIF-4 structure was generated by structure relaxation of a fully loaded ZIF-4 at 1 GPa, 303 K followed by removal of the guest pentane molecules.

To provide another way to visualize the shape and connectivity of the pores of ZIF-4, the ZIF-4 crystal structure from Park et al. [3] was fully loaded with pentane at 1 GPa (1 molecule per cage, 8 cages per unit cell) and geometry-optimized using the DREIDING force field in Materials Studio [45], causing all of the windows to open. We then removed the guess molecules, and the “opened-up” structure was analyzed using Poreblazer [47] where it was discretized onto a uniform grid (0.2 Å spacing used here) and a probe atom (2 Å diameter used here) was placed at every grid point where there was no overlap with the framework atoms. The results, a typical pore junction and the four cages connected to it, are visualized in Figure 6b. The pore size distributions of the original ZIF-4 structure [3] and the “opened-up” structure are shown in Figure 6a.

### 4.2. Molecular Simulations

Single component adsorbed configurations of C2–C5 *n*-alkanes in ZIF-4 were generated using grand canonical Monte Carlo (GCMC) simulations. The structure of ZIF-4 was taken from Park et al. [3], and the framework was kept rigid throughout the simulations, using a 2 × 2 × 2 supercell (a = 30.790 Å, b = 30.615 Å, c = 36.852 Å, α = β = γ = 90°). The interactions of the framework atoms with the *n*-alkanes and the non-bonded interactions of alkane molecules among themselves were modeled using Lennard-Jones interactions. Lennard-Jones parameters for the framework atoms were taken from the DREIDING force field [48], and the alkanes were modeled using the TraPPE [49] united-atom force field. A cut-off radius of 12.8 Å was used for the Lennard-Jones interactions, with the inclusion of tail corrections. Electrostatics are not included in the calculations. Simulations were run for a total of 10^5^ cycles, where one cycle on average corresponds to one Monte Carlo move attempted for each molecule in the box. Configurational bias reinsertions and cut-and-regrow moves were used alongside the usual insertion, deletion, rotation, and translation moves to improve the sampling. We used the in-house simulation package RASPA [50] for the GCMC simulations.

### 4.3. Synthesis of ZIF-4

ZIF-4 was synthesized by a modified procedure based on the work of Park et al. [3]. Therefore 2.4 g of imidazole and 3.64 g of zinc nitrate hexahydrate where dissolved in 120 mL of dimethylformamide in a 250 mL round-bottom flask. The solution was heated to 403 K and the temperature was kept for 48 h under stirring. After cooling down the solution was filtrated. The obtained particles were centrifuged three times with 35 mL of ethanol.

### 4.4. Adsorption Isotherms

**Figure 7:**
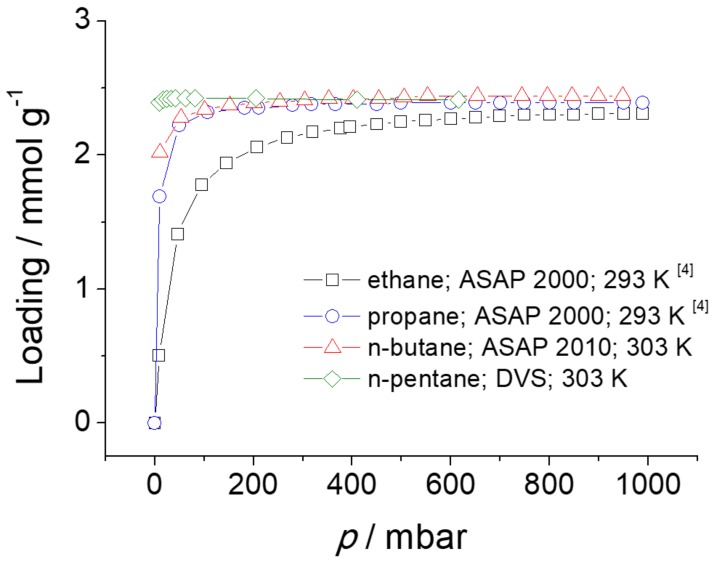
Adsorption isotherms of *n*-alkanes in ZIF-4.

The adsorption isotherms of ethane, propane, *n*-butane, and *n*-pentane in ZIF-4 are shown in Figure 7. The data are from literature (ethane and propane) [4] and the results of measurements with the volumetric adsorption apparatus ASAP 2010 from Micromeritics (*n*-butane) and the gravimetric technique of dynamic vapor sorption (DVS) (*n*-pentane).

The single component adsorption isotherm of *n*-butane was measured volumetrically at 303 K up to a pressure of 1 bar. On the other hand, the single component adsorption isotherm of *n*-pentane (with a purity ≥ 99%, purchased from Carl Roth) was determined gravimetrically in a DVS-ET (Dynamic Vapor Sorption-Elevated Temperature) from Surface Measurement Solutions. The sample chamber is constantly purged with 200 mL min^−1^ of gas. As purge gas nitrogen is used. To adjust a certain partial pressure of *n*-pentane a fraction of the nitrogen stream is saturated with *n*-pentane and then mixed with the unsaturated fraction of the nitrogen stream.

**Figure 8:**
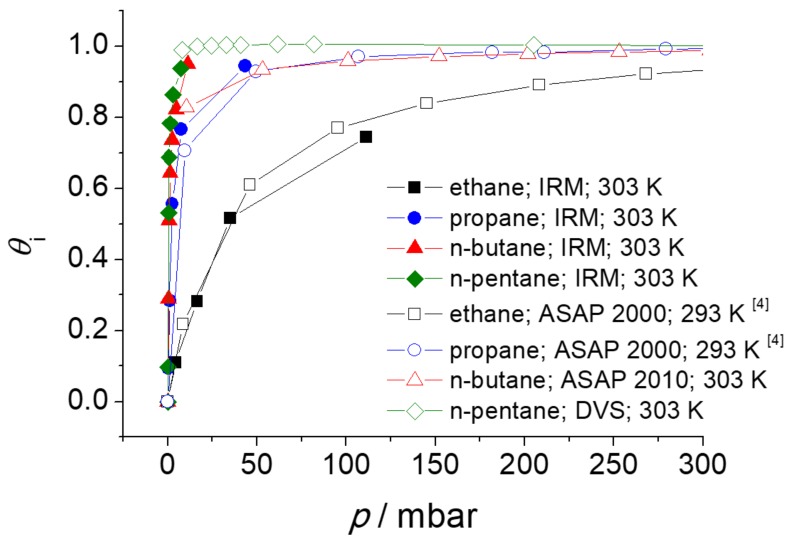
Adsorption isotherms (in relative units, i.e., the fractional pore occupancy $\theta_{i}$) of *n*-alkanes in ZIF-4, obtained with IRM, volumetric adsorption apparatus and gravimetric dynamic vapor sorption (DVS).

The absolute molar loadings (mmol g^−1^) were converted to the fractional pore occupancies using Equation (3). Since the IR signals from the IRM were measured in arbitrary units, the conversion was necessary to compare the adsorption isotherms in Figure 7 with the adsorption isotherms obtained with IRM. The comparison is shown in Figure 8.

It was detected that the adsorption properties of ZIF-4 changed over time (see details in Section 4.7). Once the sample is aged, the *n*-butane adsorption at low pressures becomes lower and also ZIF-4 exhibits sorption hysteresis for *n*-butane in the pressure range between 0 and ca. 200 mbar. Such hysteresis was not observed at the time of IR measurement, but occurred only when the volumetric sorption measurement of *n*-butane with ASAP 2010 was carried out at a later time. Hence, the desorption branch of *n*-butane has been taken in Figure 7 and Figure 8, assuming that there is no sorption hysteresis, especially in fresh ZIF-4. Further investigation is needed to understand the origin of the sorption hysteresis.

### 4.5. Sample Activation

Prior to each IR measurement, the material was activated by the following procedure. Several agglomerates of ZIF-4 crystals were placed on the bottom window of a cylindrical, optical-quality quartz glass cell. Then, the cell was heated to a temperature of 423 K at a heating rate of 1 K min^−1^ under vacuum (<10^−5^ mbar) and left there for at least 17 h. After the activation, it was cooled down to the desired temperature, i.e., 303 K, 348 K or 373 K, and the adsorption experiments were commenced.

For the adsorption measurements of ethane, propane and *n*-butane using the volumetric adsorption setup, ZIF-4 particles were prior activated at 423 K under vacuum for at least 5 h. In the case of *n*-pentane, ZIF-4 particles were activated in pure nitrogen atmosphere at 453 K for 4 h in the sample chamber of the DVS instrument.

### 4.6. IRM Experimental Setup

IRM experiments were performed on an IR microscope (Hyperion 3000, Bruker Optik GmbH, Ettlingen, Germany) connected to a vacuum FT-IR spectrometer (Vertex 80v, Bruker Optik GmbH, Ettlingen, Germany) with a polychromatic IR source. The microscope is equipped with a conventional single-element MCT detector (mercury cadmium telluride) which can set the area of interest by adjusting the size and the position of a rectangular aperture. Hence, the averaged concentration of guest molecules over a selected agglomerate or even a single crystal can be measured separately.

After the activation procedure described above, the IR optical cell (Infrasil^®^, Starna GmbH, Pfungstadt, Germany; window inner diameter 19 mm, path length 5 mm) containing the activated agglomerates of ZIF-4 crystals was mounted on the motorized platform in the focus of the IR microscope. The cell was connected to a static vacuum system (stainless steel, ¼” Swagelok tubing and valves) consisting of a pumping station (HiCube 80 Classic, Pfeiffer Vacuum GmbH, Asslar, Germany), cylindrical gas reservoirs filled with guest molecules, and pressure transducers which can measure the pressure in the range of 10^−6^ to 10^4^ mbar.

**Figure 9:**
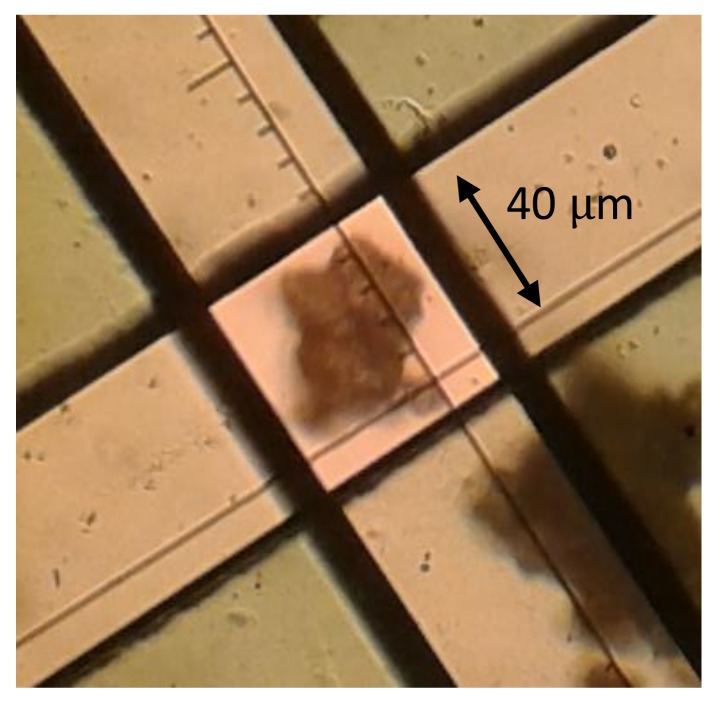
ZIF-4 agglomerate, which has been subject to IR measurements, under the microscope. The black square in the middle is the measurement window.

In the present work, only an agglomerate of ZIF-4 crystals was investigated without a carrier gas or a continuous gas flow, and the size of the agglomerate under study was approximately 40 µm (Figure 9). The pressure step changes in the gas phase surrounding the ZIF-4 agglomerate were accomplished within a fraction of a second after opening the entrance valve between the IR optical cell and the gas reservoir containing the guest molecules, and the final gas phase pressures were effectively maintained at the desired values due to a relatively large volume of the gas reservoir compared to the volume of the IR optical cell. In the case of two-component experiments, two species, namely *n*-pentane and deuterated *n*-butane, were prepared in separate gas reservoirs at the desired pressures. By opening the connecting valve between the two reservoirs, the binary mixture was prepared and then, it was introduced to the IR optical cell.

The data in Figure 1 were obtained from time-resolved uptake measurements with a spectral resolution of 16 cm^−1^ and a temporal resolution between 0.6 and 3.7 s (depending on the number of scans) using the single element detector. The integration of the time-resolved spectrum over the characteristic IR band of each guest molecule (C-H vibration band near 2950 cm^−1^) resulted in an integral “uptake curve”. By fitting the uptake curve with Equation (1) the transport diffusivity was calculated. In the two-component experiments, on the other hand, the integration was carried out over not only the C-H band but also the C-D band to distinguish the uptake of the deuterated *n*-butane from that of *n*-pentane.

### 4.7. Sample Aging

**Figure 10:**
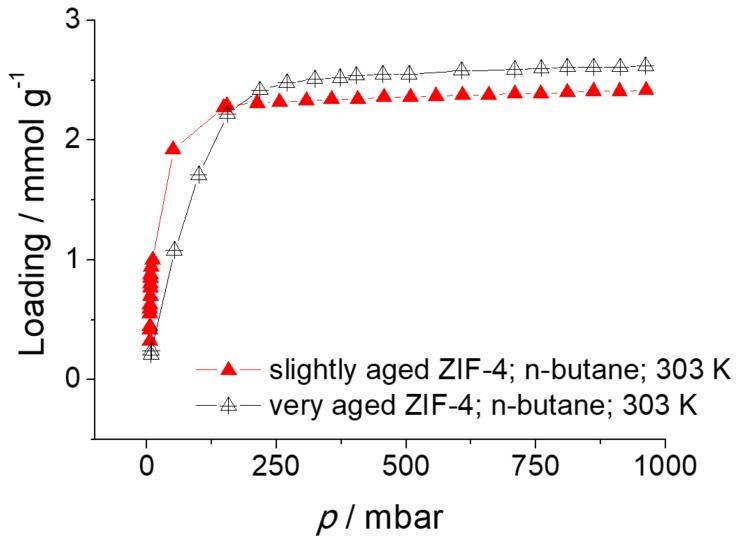
Adsorption isotherms of *n*-butane in slightly and very aged ZIF-4 at 303 K.

**Figure 11:**
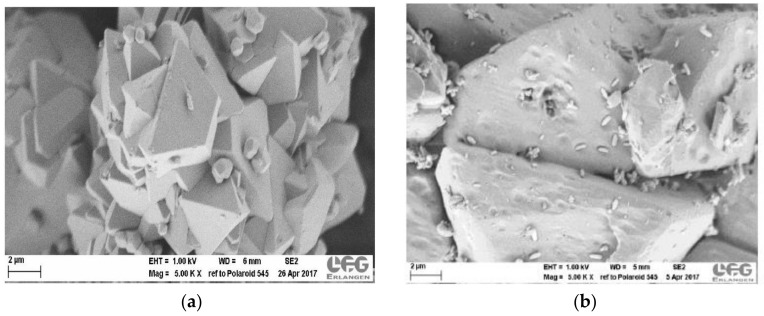
SEM images of (**a**) slightly aged ZIF-4 and (**b**) very aged ZIF-4.

Figure 10 shows the adsorption isotherms of aged ZIF-4 samples, volumetrically measured with ASAP 2010 at 303 K. The relatively fresh ZIF-4 exhibits higher adsorption at low pressure, i.e., steeper initial slope, than the very aged ZIF-4. Scanning electron microscopy (SEM) images of the two samples are also provided in Figure 11. It is clear that there is difference in the surface between the two: the slightly aged ZIF-4 shows a smooth surface without any small white particles while the very old ZIF-4 exhibits shallow craters on the surface, on which white particles are attached. It is seen that the ZIF-4 surface changes with time, which might affect the overall adsorption properties of the ZIF-4. This may have led to the minor discrepancy in adsorption isotherms between the ZIF-4 measured with IRM (fresh ZIF-4) and the one measured with ASAP 2010 (aged ZIF-4) shown in Figure 8.

## 5. Conclusions

The pattern of guest diffusivities increasing rather than decreasing with increasing molecular sizes is one of the remarkable peculiarities of microporous host-guest systems. This phenomenon is generally referred to as the “window effect”, following the nomenclature chosen in a first publication dealing with this effect. Though the anomaly reported there is today known to be an artefact of data analysis caused by unjustified simplifications, most convincing evidence has meanwhile been accumulated by molecular simulation so that there is no doubt about the possibility of their existence. However, deviating from the clear message of theoretical prediction, robust experimental data for deepening this phenomenon are so far scarcely available. Novel options for such studies have been opened up with the advent of new generations of microporous materials. This does, notably, concern the family of metal-organic frameworks (MOFs) with unprecedented options for an essentially arbitrary variation of the pore sizes in relation to the size of the guest molecules.

We have reported here first systematic diffusion measurements with short chain length *n*-alkanes in MOFs of type ZIF-4. Following the chain length dependence of the diffusivities from ethane to *n*-hexane, the *n*-pentane diffusivity is found to exhibit a pronounced maximum. Owing to the potential of IR microscopy, measurements could be performed with single adsorbent particles and over small pressure and concentration steps. Corruptions of the measurement as typical for some of the early uptake measurements could thus be avoided. Experimental evidence was, moreover, also confirmed by the performance of two-component measurements where the rate of *n*-pentane uptake was found to significantly exceed that of *n*-butane. The message of the experimental diffusion studies is, finally, also supported by the results of grand canonical Monte Carlo simulations. By considering the influence of spatial confinement within the cage system of ZIF-4 on molecular configurations, *n*-pentane may be concluded not to have enough room to fit comfortably in the ZIF-4 cages which might be easily correlated with a tendency towards higher jump rates between adjacent cages.

In future studies, we may perform release measurements in ZIF-4 to study whether the non-monotonic behavior of the *n*-alkane diffusivities is still present during the transient desorption, along with improved GCMC simulations where the framework flexibility of ZIF-4 is considered. A further investigation on the origin and the effects of the sample aging phenomenon will be also considered.

## Figures and Tables

**Table 1:** Enthalpy of adsorption of C_2_–C_5_ *n*-alkanes in ZIF-4 at 2 bar and 303 K calculated via GCMC simulations in a rigid framework.

Alkane	−Δ*H*_ads_ (kJ moL^−1^)
ethane	−38.4 ± 2.01
propane	−44.9 ± 7.00
*n*-butane	−46.2 ± 7.57
*n*-pentane	−42.4 ± 6.83

**Table 2:** End-to-end distances of C_2_–C_5_ *n*-alkanes in their fully stretched conformations. The methyl radii on both ends are included in the length.

Alkane	Fully Stretched End-To-End Distance (Å)
ethane	5.29
propane	6.83
*n*-butane	7.67
*n*-pentane	8.91

**Table 3:** ZIF-4 properties calculated using Zeo++ [46] with a 2.4 Å probe.

Property	Value
Density	1.22 g cc^−1^
Accessible Surface Area	771 m^2^ cc^−1^
Accessible Volume	1776 m^3^ g^−1^
Void Fraction	0.56 (zero probe)
Channels	1 (3 dimensional)
Largest Cavity Diameter	5.14 Å
Pore Limiting Diameter	2.45 Å
